# Effect of Nanodiamond Sizes on the Efficiency of the Quasi-Specular Reflection of Cold Neutrons

**DOI:** 10.3390/ma16020703

**Published:** 2023-01-11

**Authors:** Alexei Bosak, Marc Dubois, Ekaterina Korobkina, Egor Lychagin, Alexei Muzychka, Grigory Nekhaev, Valery Nesvizhevsky, Alexander Nezvanov, Thomas Saerbeck, Ralf Schweins, Alexander Strelkov, Kylyshbek Turlybekuly, Kirill Zhernenkov

**Affiliations:** 1 European Synchrotron Radiation Facility, 71 Av. des Martyrs, F-38043 Grenoble, France; 2 Clermont Auvergne INP, Université Clermont Auvergne, CNRS UMR6296, 24 Av. Blaise Pascal, F-63178 Aubière, France; 3 Department of Nuclear Engineering, North Carolina State University, Raleigh, NC 27695, USA; 4 Frank Laboratory of Neutron Physics, Joint Institute for Nuclear Research, 6 Joliot Curie, Ru-141980 Dubna, Russia; 5 Institut Max von Laue—Paul Langevin, 71 Av. des Martyrs, F-38042 Grenoble, France; 6 Faculty of Physics and Technology, L.N. Gumilyov Eurasian National University, Satpayev Str. 2, Astana 010000, Kazakhstan; 7 The Institute of Nuclear Physics, Ministry of Energy of the Republic of Kazakhstan, Ibragimova Str. 1, Almaty 0500032, Kazakhstan; 8 JCNS at Heinz Maier-Leibnitz Zentrum (MLZ), Forschungzentrum Jülich GmbH, 1 Lichtenbergstrasse, D-85748 Garching, Germany

**Keywords:** quasi-specular reflection, nanodiamonds, nanopowder, reflectors of slow neutrons, albedo, fluorination

## Abstract

Nanomaterials can intensively scatter and/or reflect radiation. Such processes and materials are of theoretical and practical interest. Here, we study the quasi-specular reflections (QSRs) of cold neutrons (CNs) and the reflections of very cold neutrons (VCNs) from nanodiamond (ND) powders. The fluorination of ND increased its efficiency by removing/replacing hydrogen, which is otherwise the dominant cause of neutron loss due to incoherent scattering. The probability of the diffuse reflection of VCNs increased for certain neutron wavelengths by using appropriate ND sizes. Based on model concepts of the interaction of CNs with ND, and in reference to our previous work, we assume that the angular distribution of quasi-specularly reflected CNs is narrower, and that the probability of QSRs of longer wavelength neutrons increases if we increase the characteristic sizes of NDs compared to standard detonation nanodiamonds (DNDs). However, the probability of QSRs of CNs with wavelengths below the cutoff of ~4.12 Å decreases due to diffraction scattering on the ND crystal lattice. We experimentally compared the QSRs of CNs from ~4.3 nm and ~15.0 nm ND. Our qualitative conclusions and numerical estimates can help optimize the parameters of ND for specific practical applications based on the QSRs of CNs.

## 1. Introduction

The goal of the present work is to shed more light on the phenomena occurring when materials with characteristic structural sizes of the order of the wavelength of the incident radiation intensively scatter and, under certain conditions, reflect the radiation. The corresponding interaction mechanisms of structured materials regarding the conditions for the efficient reflection of radiation, as well as the structures of the materials themselves, are of great theoretical and practical interest [1,2]. 

This phenomenon has been observed for many types of radiation and types of structured materials. In particular, scattering neutrons to fluctuate the neutron–nucleus potential of a substance is a powerful method for studying the sizes, shapes and positions of scattering centers in a substance, and the standard experimental method for studying them is small-angle neutron scattering (SANS) [3,4,5,6]. 

We studied a new class of such phenomena, the extremely intense coherent scattering of very cold neutrons (VCNs) and cold neutrons (CNs) on detonation (DND) and shock compression (SCD) nanodiamonds [7,8]. The characteristic velocity of VCNs of V_VCN_ ~50 m/s corresponds to a wavelength of λ_VCN_ ~8 nm. The characteristic velocity of CNs of V_CN_ ~500 m/s corresponds to a wavelength of λ_CN_ ~0.8 nm. The characteristic DND diameter of d_DND_ ~4–5 nm lies between these two values, whereas the SCD diameter of d_SCD_ ~15 nm (used here) is larger. 

DND powders can diffusively reflect VCNs at any angle of incidence [9,10,11,12] and can reflect CNs quasi-specularly at small angles of incidence [13,14,15]. The potential applications of these phenomena in neutron technologies and experiments [16,17,18,19,20] and the properties of such reflectors [21,22,23,24,25,26,27,28] are being actively studied. In addition, DND reflectors are being integrated into standard neutron simulation packages [28,29,30,31]. The efficiency of reflectors has been improved by removing hydrogen (H) from the DND surface using fluorination [32,33], accompanied by destroying clusters in the DND powder [34] and, in the case of the diffuse reflection of VCNs, by choosing the optimal DND size [35].

In the case of the quasi-specular reflection (QSR) of CNs, the angular distribution of reflected neutrons forms a peak near the specular reflection angle. For the possible use of QSR, not only is the probability of total reflection important, but the width of the angular distribution of reflected neutrons is as well. As the distribution becomes narrower, the reflector becomes better. In reference to ref. [35], and on the basis of our model ideas about the interaction of CNs with nanodiamonds (NDs), we assumed that it is possible to optimize the sizes of NDs to increase the efficiency of the QSR of CNs. In particular, an even narrower angular distribution of quasi-specularly reflected CN, as well as an even larger total probability of neutron reflection at relatively long wavelengths, can be obtained by increasing the characteristic size of NDs. The reasons for these assumptions are as follows. First, the characteristic angle of CN single scattering on an ND particle Δθ_1_ [36] is equal to

Δθ_1_~λ_CN_/2πd_ND_, (1)

which gives ~2° for the characteristic values above. Because the QSR of CN is the result of one or more scattering events on an individual ND, the characteristic angle of QSR, Δθ_QS_, is of the same order: Δθ_QS_~Δθ_1_. Therefore, it is expected that an increase in the ND size d_ND_ leads to a decrease in the characteristic QSR angle Δθ_QS_. Second, an increase in the size d_ND_ greatly increases the cross section of the coherent scattering of CNs on an individual ND, which might lead, under some conditions, to an increase in the total probability of QSR [9]. 

However, the appearance of these two positive effects is associated with a negative one. The probability of the QSR of CNs with relatively short wavelengths is suppressed more strongly due to the stronger effect of diffraction scattering on the ND crystal lattice. Such suppression occurs at characteristic CN wavelengths comparable to interatomic distances in a diamond crystal and below. The boundary of this range approximately corresponds to the maximum interplanar spacing in diamonds with a nonzero structure factor, which is given by the (111) plane and is equal to ~4.12 Å. The diffraction lines are broadened out due to the small value of d_ND_. 

Because several characteristic quantities of this problem have the same order of magnitude (interatomic distance, neutron wavelength, ND size and distance between neighboring ND), there are, in general, no “smallness parameters” in this problem, and its calculation “from first principles” is hardly possible. Therefore, we chose a different approach to solve it. 

Based on the conclusions from previous measurements of the QSR of CNs [14,15,23], and based on simplified models of the QSR of CNs, we chose an ND with a factor of ~3 larger than d_DND_ and compared the results with previous ones obtained in the same configuration using the standard size of DNDs. In both cases, parts of the samples were fluorinated to reduce CN losses. We compared the obtained experimental results with each other, as well as with simplified models that allowed us to understand the essence of the observations. Based on the experimental data and analysis, we made conclusions about the optimal parameters of NDs for observing and using the QSR of CNs. 

The samples used for this study are presented in Section 2. The experimental methods used in this work are explained in Section 3. The experimental results obtained are presented in Section 4, simulated in Section 5 and analyzed in Section 6. 

## 2. Samples

**Figure 1:**
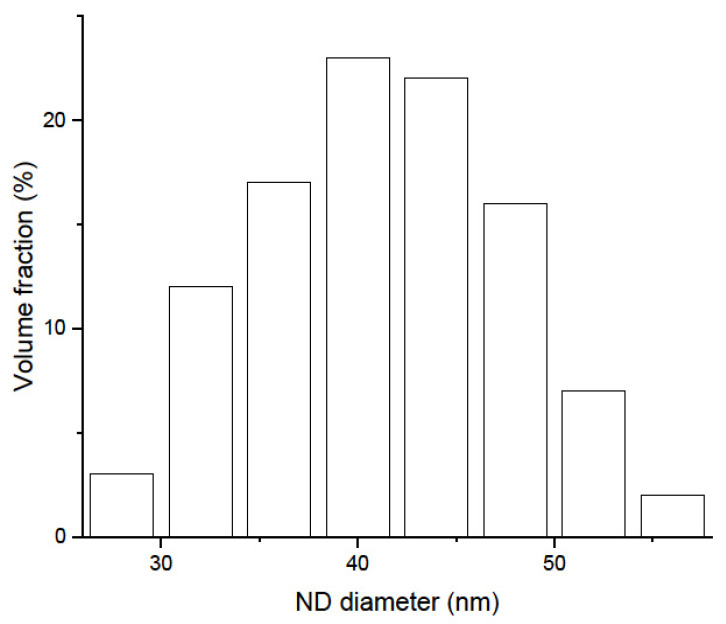
Diameter distribution of ND in the SCD powder measured by Ferran using a Beckman Coulter laser diffraction particle size analyzer. Note that the sensitivity of the method sharply decreases for small NDs (below ~30 nm), and the method should not be used in this range.

To study the effect of the ND size on the efficiency of the QSR of CNs, we used four types of ND samples:

1. DND: DND powder produced by FSUE “RFNC-VNIIF” using the detonation method, with a mean diameter of diamond cores of ~4.3 nm (when weighted over particles). The spread of DND diameters is approximately in the range of 2–10 nm. Powders of this type were also used in refs. [11,12,14,15,23,27,28,34,35]. The sample density is 0.29 g/cm^3^.
2. F-DND: DND powder fluorinated according to the procedure described in refs. [32,33]. Fluorination replaces H and removes carbon (C) in the *sp*^2^ hybridization found in ND shells. However, it does not change the size of the diamond cores of the ND. As a result, neutron losses are dramatically reduced, and the scattering properties of ND remain virtually unchanged. The sample density is 0.29 g/cm^3^.
3. SCD: ND powder produced by “Ferran”, Altai, using the shock compression (SCD) method. A preliminary estimation of the ND diameters (Figure 1) was performed by Ferran using a Beckman Coulter laser diffraction particle size analyzer. This method is insensitive to ND diameters below ~30 nm. Therefore, the result is biased towards larger diameters and thus larger ND masses. In order to better estimate the unbiased value, we used a method related to Sherrer’s equation on X-ray diffraction data (Section 4.2) and obtained a mean diameter of ~15 nm. The sample density is 0.39 g/cm^3^.
4. F-SCD: Fluorinated SCD powder. The fluorination procedure is presented in Section 3.1, and the results of fluorination are described in Section 3.2 and Section 4.1. The sample density is 0.39 g/cm^3^.

## 3. Experimental Methods

### 3.1. Fluorination of SCD

The fluorination of DNDs is fully described in refs. [32,33]. The procedure of SCD fluorination is developed in the framework of this study; therefore, it is described in detail. 

SCDs were fluorinated using static conditions in a closed nickel reactor (passivated with NiF_2_). SCDs were placed onto passivated nickel supports inside the reaction vessel. Prior to fluorine gas insertion, the reactor was evacuated to form a primary vacuum (~10^−2^ mbar) and was heated for 2 h at a temperature of 200 °C in order to remove water molecules adsorbed on the SCD surface. Pure fluorine gas (~99.9% purity) was then added with a high flux (~200 mL/min), and a heating rate of 20 °C/min was applied to reach a temperature of 500 °C. This final temperature was stabilized for 12 h. If the pressure inside the reactor increased to 1.2 bar (because of the heating or reaction), the valve between the reactor and soda lime trap was opened, and the pressure was stabilized to 1 bar. 

Such drastic conditions are unusual for graphitic carbonaceous materials because they may result in exfoliation. In the present case, the aim was the decomposition of *sp*^2^ C shells located on the diamond core, whereas diamond-type C would not react. Fast heating and fluorine addition favor this decomposition with CF_4_, C_2_F_6_ and the other biggest fluorinated fragment released from the nanoparticles. 

After cooling to room temperature, the reactor was flushed with nitrogen flow (600 mL/min) for 1 h to remove unused fluorine, HF and decomposition products (CF_4_, C_2_F_6_, etc.). Because of the quantity of ND of less than 10 g, F_2_ gas was in excess for the completion of the purification process. The resulting fluorinated samples of DND and SCD are denoted as F-DND and F-SCD, as mentioned above. 

### 3.2. Physicochemical Characterization of F-SCD

FTIR experiments were conducted with a Nicolet 6700 FT-IR spectrometer both in the Attenuated Total Reflection (ATR) and transmission modes. For each spectrum, 128 scans with a 4 cm^−1^ resolution were collected between 4000 and 450 cm^−1^. 

^1^H, ^19^F and ^13^C solid-state NMR experiments were performed using a 300 MHz Brucker Avance spectrometer at room temperature. A cross polarization (CP)/magic-angle spinning (MAS) NMR probe operating with 4 mm rotors was used at 15 kHz, 15 kHz and 10 kHz spinning rates for ^1^H, ^19^F and ^13^C measurements, respectively. A probe (Bruker) with fluorine decoupling on a 4 mm rotor was used. For MAS spectra, a simple sequence was performed with a single π/2 pulse length of 4.0, 4.0 and 3.5 μs for ^1^H, ^19^F and ^13^C, respectively. ^13^C NMR was performed at a frequency of 73.4 MHz, and tetramethylsilane (TMS) was used as the reference. ^19^F MNR was carried out with a frequency of 282.2 MHz, and the spectra were externally referenced with CF_3_COOH and then with CFCl_3_ (δ_CF3COOH_ = −78.5 ppm/CFCl_3_). Quantitative ^19^F NMR using polytetrafluoroethylene (PTFE) as an internal reference was carried out to determine the content of each C-F bond. For this aim, the recycling time (D_1_) was fixed according to the longer spin-lattice relaxation time T_1_, i.e., for PFTE. Therefore, D_1_ was 25 s (3 s is enough for conventional fluorinated carbons). 

Nitrogen adsorption isotherms were measured at a temperature of 77 K using a Micromechanics ASAP 2020 automatic apparatus. Before measurements, samples were pre-treated under a secondary vacuum at a temperature of 300 °C for 2 h for the sufficient removal of adsorbed impurities.

### 3.3. XRD Size Characterization

X-ray diffraction (XRD) data were collected using the ID28 diffractometer at ESRF [37] with a PILATUS3 1M area detector (at the X-ray wavelength λ = 0.784 Å). The samples were packed in quartz capillaries with a diameter of ~200 μm. The data were reduced using SNBL Toolbox [38] and Dioptas [39] software. The instrumental resolution was evaluated using the LaB_6_ standard. The observed diffraction lines corresponded to the diamond lattice. They showed significant size-related broadening and Lorentzian-like shapes, indicating a broad distribution of sizes. Thus, the direct application of Sherrer’s equation may not have been reliable. Instead, the line shape was analyzed by combining FW1/5M and FW4/5M, as described in ref. [40], within the model of the Gamma distribution of nanoparticle sizes.

### 3.4. Small-Angle Neutron Scattering and Quasi-Specular Neutron Reflection

Small-angle neutron scattering (SANS) on DND/F-DND samples was performed with the D11 instrument [41] at the Institut Laue-Langevin (ILL) in Grenoble, France. More information can be found in ref. [28]. 

**Figure 2:**
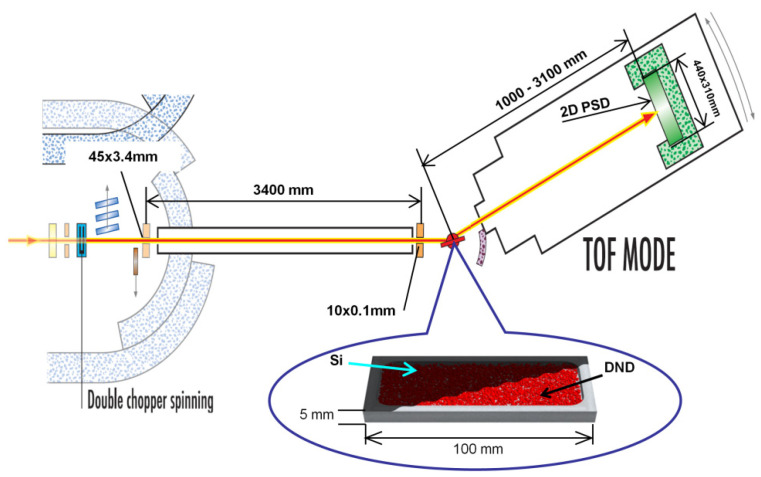
Top view scheme of measurements of the QSR of neutrons from the powder of NDs. The circled magnification shows a sample of ND powder (red) in a sample container. The double chopper periodically interrupts the incident neutron beam, thus providing a pulsed neutron spectrum for time-of-flight measurements. Various devices in front of the sample provide background suppression, neutron transport and neutron beam collimation. A position-sensitive ^3^He detector (2D PSD) is behind the sample. The arrow illustrates a neutron quasi-specularly reflected from the sample.

The QSR of neutrons was studied using the D17 [42] instrument. The neutron spectrum was measured using the time-of-flight method; the range of wavelengths was 2–25 Å. The neutron incidence angles were 1°, 2° and 3°. The measurement scheme of the D17 instrument is shown in Figure 2. The ND sample volume size was 100 × 50 × 5 mm^3^; the sample was oriented vertically, meaning that 100 mm was oriented along the neutron beam, and 50 mm was aligned vertically. The neutron beam was shaped by two diaphragms located at a distance of 3.4 m from each other. The beam width was 0.1 mm, and the angular spread in the horizontal plane was <0.1° for all neutron wavelengths. The beam height in the vertical direction was 1 cm, and the beam angular divergence in the vertical plane reached several degrees and depended on the neutron wavelength. The experimental data coming from the detector were integrated along the detector vertical. Therefore, we could analyze the angular distribution of neutrons reflected from the sample only in the horizontal plane (the plane in Figure 2 perpendicular to the sample surface). The detector had an active height and width of 473 × 250 mm^2^ and was placed 1 m from the sample to cover the largest possible angle. 

## 4. Experimental Results

### 4.1. Physicochemical Characterization of F-SCD

**Figure 3:**
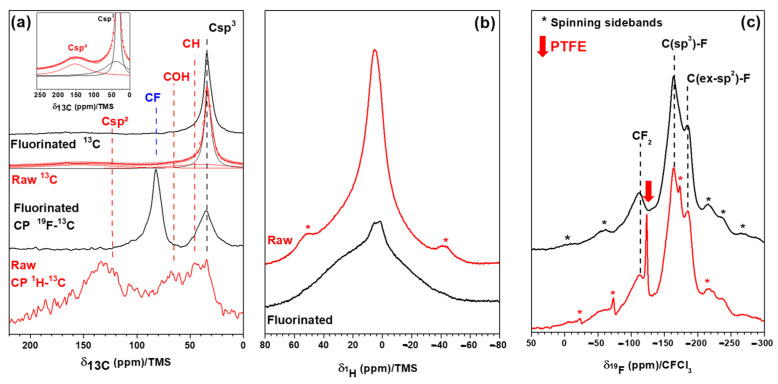
(**a**) ^13^C MAS (10 kHz) spectra of raw and fluorinated SCD (^1^H→^13^C and ^19^F→^13^C CP were also used for raw and fluorinated samples); (**b**) ^1^H MAS (14 kHz); (**c**) ^19^F MAS (14 kHz) spectra.

The ^13^C MAS spectrum of raw SCD (Figure 3a) exhibits a broad line centered at 120 ppm and was then assigned to amorphous *sp*^2^ C. Their high content is estimated at ~29% using the fit of the spectrum (see the insert in Figure 3a), which is significantly higher than that for DND because this resonance line was not observed in refs. [32,33]. ^1^H to ^13^C cross-polarization acquisition also evidences those *sp*^2^ C, together with diamond-type (C_D_), C-OH and C-H, according to the lines at 139, 120, 70 and 45 ppm, respectively [27,32,43,44,45]. However, only C_D_, OH and C-H are observed for DND with CP [32,43]. The line of diamond C_D_ is observed in the single MAS and CP-MAS experiments. Contrary to DND, *sp*^2^ C cannot be fully removed with fluorination for SCD. After this treatment, the content of *sp*^2^ C decreases to ~19%. Due to weak interactions with the neighboring C-F bonds, the chemical shift of *sp*^3^ C was 42 ppm for F-SCD rather than 38 ppm in pure diamond. Regardless of the type of diamond, covalent C-F bonds are formed during fluorination, as evidenced by the line at 84 ppm. The ^19^F→^13^C CP condition evidences those better bonds. Because those measurements are not quantitative, the differences cannot be explained (C-F line is twice the C_D_ one for F-SCD, whereas the intensity is equal for F-DND).

The content of H-containing groups (C-H and COH) is significantly higher in SCD than that in DND [32,43] (Figure 3b). For DND, a weak line is observed in addition to the ones of an empty rotor, as seen in refs. [32,43]. The H content in F-SCD decreases significantly after fluorination, and the line almost disappears.

**Figure 4:**
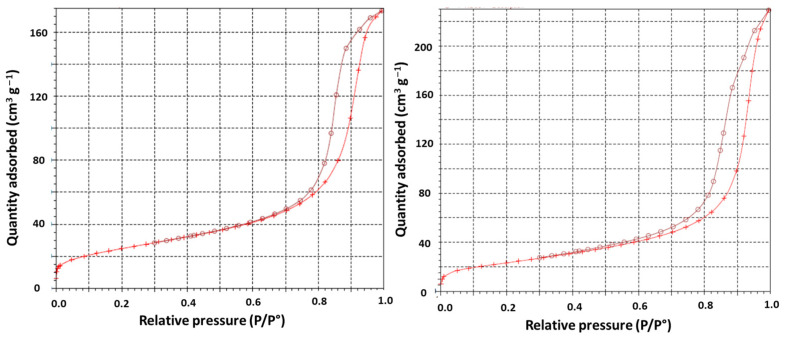
Adsorption and desorption isotherms of SCD (**left**) and F-SCD (**right**).

The fluorination of SCD results in two types of C-F bonds: fluorine bonded on *sp*^3^ C atoms, similar to F-DND [27,32,43,45] (−164 ppm, denoted by C(*sp*^3^)-F in Figure 3c), but also C-F bonds in fluorinated amorphous carbons (−190 ppm, C(ex-*sp*^2^)-F. Ex-*sp*^2^ means *sp*^2^ hybridization before fluorination. The latter C-F bonds exhibit δ_19F_ similar covalent (CF)_n_-type graphite fluoride, i.e., −190 ppm. The intensity of the line at 120 ppm that is assigned to the CF_2_ groups is higher for F-SCD than that for F-DND, in accordance with the non-completion of decomposition under the F_2_ gas of the amorphous *sp*^2^ shell. The number of perfluorinated sheet edges (with CF_2_) is low but not negligible in the residual fluorinated layer for F-SCD. Because of the very low extent of the *sp*^2^ C shell on the DND surface, the amount of C(ex-*sp*^2^)-F bonds (δ_19F_ = −190 ppm) resulting from this shell is low, and the fluorinated carbons are formed mainly from the conversion of C-OH and/or C-H groups [27,32,43]. Quantification using the fit of ^19^F spectra with four Lorentzian lines (C(*sp*^3^)-F, C(ex-*sp*^2^)-F, CF_2_ of F-DND or F-SCD, and CF_2_ of PTFE as an internal reference) allows a chemical composition of CF_0.097_ to be found for F-DND, with two types of C-F bonds: CF_0.079±0.005_ for C(*sp*^3^)-F and CF_0.018±0.005_ for C(ex-*sp^2^*)-F. The ratio C(*sp^3^*)-F/C(ex-*sp^2^*)-F is then equal to 81/19 [27]. A ratio of 76/24 and the total composition of CF_0.080±0.005_ are found for F-SCD. The differences in the ratio and fluorine content may be related to the specific surface area. The Brunauer–Emmett–Teller (BET) surfaces are 281 [43] and 88 m^2^·g^−1^ for DND and SCD, respectively. More of the surface is accessible to fluorine gas, explaining higher F/C ratios in F-DND. However, the *sp*^2^ C is not accessible to F_2_ in SCD. The isotherm and pore size distribution curves are nearly the same for DND and SCD (Figure 4 for SCD and ref. [43] for DND). As the porosity is essentially inter-particular, the shapes of the N_2_ isotherms at a temperature of 77 K are type IV isotherms, which are typical for mesoporous materials according to the classification of Brunauer, Deming, Deming and Teller (BDDT) [46]. The hysteresis loop is of the H2 type, related to interconnected mesopores [47]. Neither the BET surface nor the pore size distribution is changed by the fluorination. The shapes of the isotherms are close, and the BET surface is 84 m2·g^−1^ after fluorination for F-SCD. 

**Figure 5:**
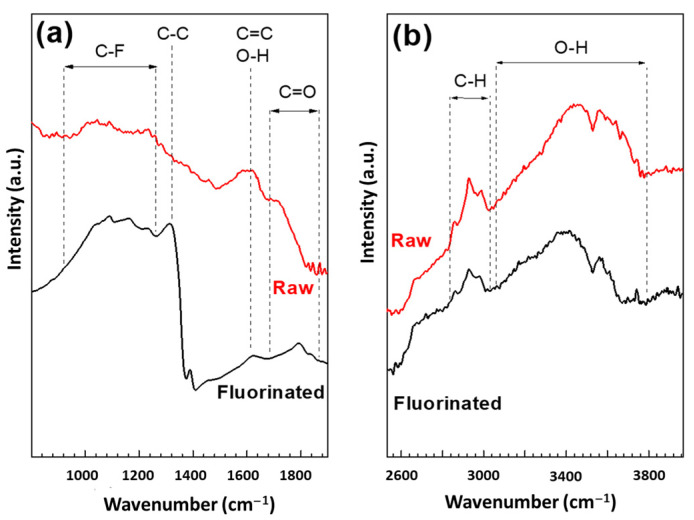
IR spectra of raw and fluorinated SCD in the two ranges of interest (**a**,**b**).

Infrared spectroscopy data confirm the dual nature of C-F bonds because of the broadening of the vibration band of C-F in the 900–1300 cm^−1^ range (Figure 5). For raw SCD, although the spectrum is less defined because of the conductive carbonaceous shell on the diamond core (as seen by NMR), the assignments are similar to that of DND [32,43]. The broad bands in the 3280–3675 cm^−1^ (Figure 5b) range and at 1640 cm^−1^ (Figure 5a) are related to O-H stretching and bending vibrations, respectively. C-H can also be underlined by the presence of the line at 2900 cm^−1^ [32,43,48,49,50,51]. The band of the C-C vibration in the diamond core at 1325 cm^−1^ is difficult to observe for SCD. Contrary to the case of DND [27,32,43], the completion of the removal of hydrogenated groups is not achieved because the corresponding bands are still present after fluorination but have attenuated intensities. 

As a partial conclusion for the physicochemical properties, F-DND and F-SCD exhibit similarities in fluorine content and in the dual nature of the C-F bonds but also differences because F-SCD contains residual *sp*^2^ C and H, which are likely not accessible for fluorine gas, contrary to F-DND. Nevertheless, the main difference is the size of the diamond core, i.e., 4–5 nm and around 15 nm for DND and SCD, respectively. The fluorination of nanodiamonds does not significantly change the crystallite size [27,32].

### 4.2. XRD Size Characterization

**Figure 6:**
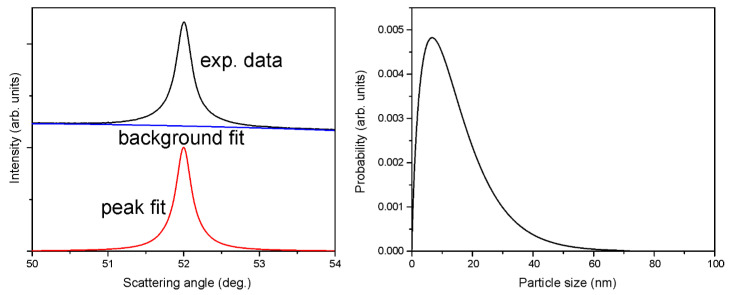
XRD results for the SCD sample. (**Left**): 400 diffraction line shape and its fit. The noise level is comparable to the line thickness. (**Right**): Evaluated ND size distribution function [40].

The XRD results for the SCD sample are shown in Figure 6. The results for DND and F-DND samples were the same as reported in ref. [35] and other previous publications. 

Diffraction line 400 was used for the evaluation of the ND size distribution following the procedure related to the Scherrer formula, as detailed in ref. [40]. Thus, the obtained parameters were <D> ≈ 15 nm and σ ≈ 11 nm. Upon weighting the size probability distribution (Figure 6) by ND particle mass, <D^4^>/<D^3^>, we obtained ~40 nm, in agreement with the preliminary estimation of the Ferran company, as shown in Figure 1.

### 4.3. Quasi-Specular Neutron Reflection

**Figure 7:**
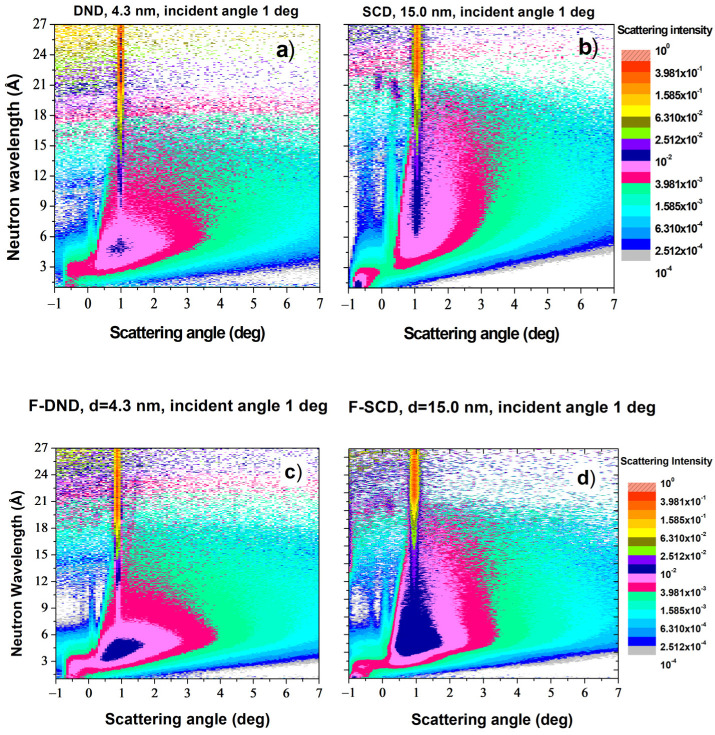
Probability of neutron scattering from the surface of ND samples as a function of the neutron wavelength (vertical axis) and the scattering angle in the direction perpendicular to the plane of the sample (horizontal axis). Different colors are scaled to the proportion of neutrons registered in the detector normalized by the incident beam flux. Samples: (**a**) DND; (**b**) SCD; (**c**) F-DND; (**d**) F-SCD. In all cases, the angle of incidence of the neutron beam onto the sample was 1°.

The probability of the QSR of neutrons is shown in Figure 7 for raw and fluorinated NDs with diamond core diameters of ~4.3 nm and ~15.0 nm. The scattered intensity is displayed as a function of the neutron wavelength and the scattering angle in the direction perpendicular to the plane of the sample surface (integrated over the angle of scattering in the sample surface plane).

The narrow vertical line of intensity with a reflection angle of 1° in Figure 7a–d corresponds to the specular reflection of neutrons from the thin Si window of the sample container, which allowed for checking the correct alignment of the sample. 

**Figure 8:**
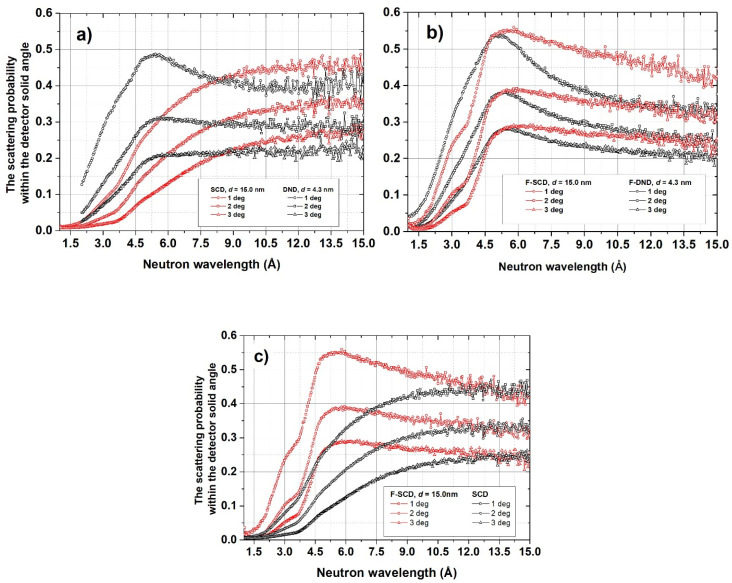
Neutron scattering probability as a function of neutron wavelength (horizontal axis) within the D17 detector acceptance. (**a**) DND and SCD samples; (**b**) F-DND and F-SCD samples; (**c**) SCD and F-SCD samples. Incident angles 1°, 2° and 3°.

Figure 8 shows the probability of neutron detection for different angles of incidence as a function of neutron wavelength integrated over all reflection angles within the angular acceptance of the detector.

**Figure 9:**
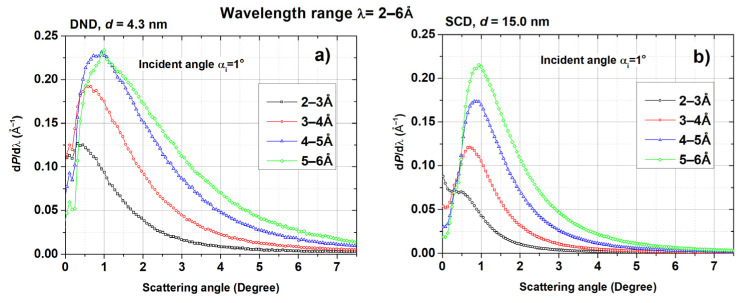

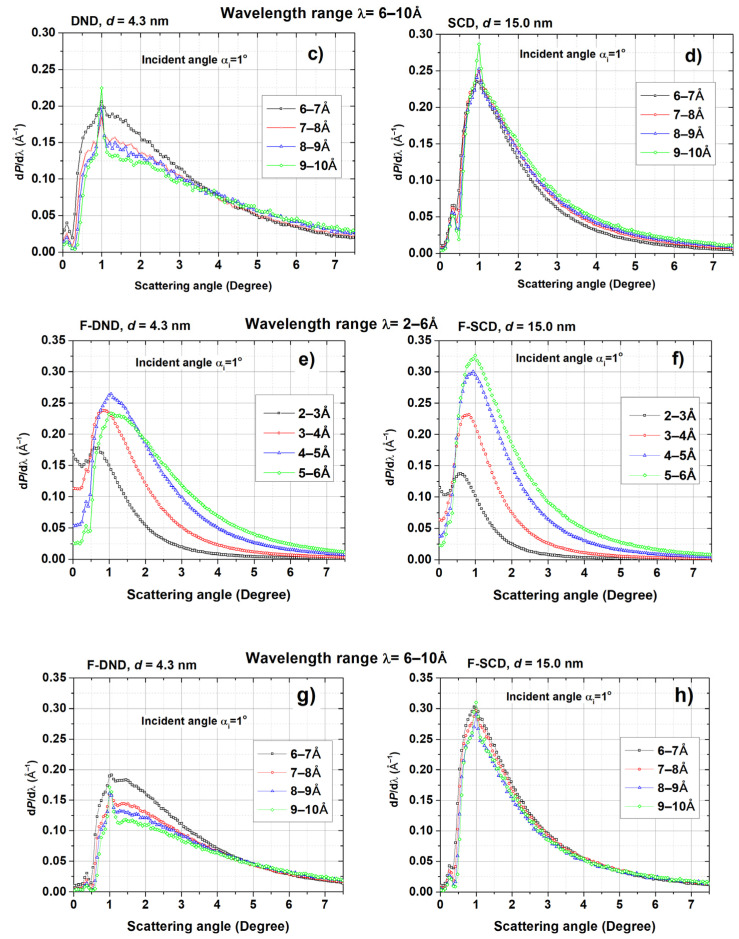
Differential probability of neutron scattering (vertical axis) as a function of the reflection angle (horizontal axis) within the angular acceptance of the D17 detector. Samples and wavelength ranges: (**a**) DND, 2–6 Å; (**b**) SCD, 2–6 Å; (**c**) DND, 6–10 Å; (**d**) SCD, 6–10 Å; (**e**) F-DND, 2–6 Å; (**f**) F-SCD, 2–6 Å; (**g**) F-DND, 6–10 Å; (**h**) F-SCD, 6–10 Å. For all cases, the angle of incidence was 1°.

The angular distribution of quasi-specularly reflected neutrons is shown in Figure 9.

## 5. Simulation of Quasi-Specular Reflection

**Figure 10:**
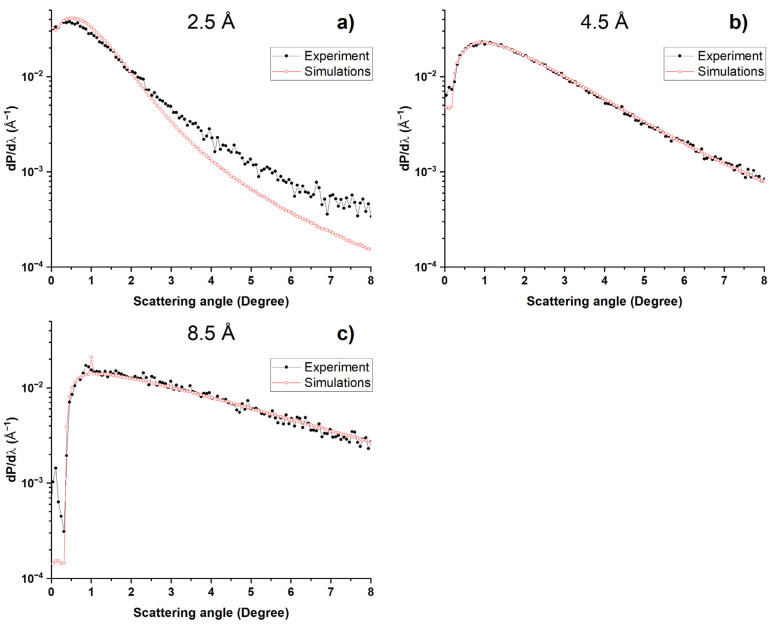
Simulated and measured angular distributions of QSR neutrons for the F-DND sample at three wavelengths: (**a**) 2.5 Å, (**b**) 4.5 Å and (**c**) 8.5 Å. In all cases, the angle of incidence was 1°.

We developed a method for simulating the transport of slow neutrons in ND powders, as described in refs. [28,34,35]. NDs are described by an optical potential. Neutron diffraction by their crystal structures is not taken into account. The implementation of this method is based on the SANS data on the ND powder under study. Such data were measured for the DND and F-DND samples but not for the SCD and F-SCD samples. To check the correctness of our calculations, we simulated the experimental geometry with the F-DND sample, taking into account the sizes and density of the sample and the detector area. The accuracy of the calculation of the total probability of reflected neutrons hitting the detector (Figure 7) was limited by the accuracy of knowing the angular divergence of the beam in the vertical direction. However, with good accuracy, we could calculate the angular dependencies of the reflected neutrons in the plane perpendicular to the sample, which are weakly affected by the beam divergence in the plane parallel to the sample surface. Figure 10 shows the results of calculations and measurements for three neutron wavelengths (2.5 Å, 4.5 Å and 8.5 Å) at an angle of incidence of 1°. 

Figure 10 shows that, for wavelengths of 4.5 Å and 8.5 Å, the results of calculations and measurements almost completely coincided. The same agreement was observed for all wavelengths greater than ~4.12 Å, for the diffraction cutoff of the (111) plane in the diamonds and for angles of incidence of 2° and 3°. For 2.5 Å, the results were different, which is explained by the effect of neutron diffraction on the crystal lattice of diamonds below the diffraction cutoff. 

**Figure 11:**
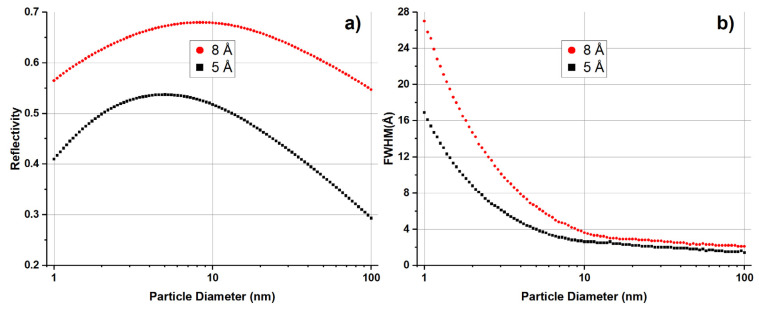
(**a**) Simulated reflectivity and (**b**) angular distribution width as a function of ND diameter. Black squares correspond to calculations for neutrons with wavelengths of 5 Å, and red circles correspond to 8 Å. The angle of incidence was set to 1° in the simulations.

These results show that our simulation was reliable for wavelengths >4.12 Å, and we could use it to predict the properties of ND reflectors for neutrons. Figure 11 shows the results of the simulation of the reflectivity and angular spread of neutrons reflected from a monodisperse ND powder as a function of ND size. The reflector had the same shape as that in the experiment (100 × 50 × 5 mm^3^). The powder density was 0.29 g/cm^3^ in all cases. In contrast to the previous case, we counted all reflected neutrons emitted from the reflector surface. The angle of incidence in the simulation was set to 1°.

Figure 11a shows that, for each neutron wavelength, there was a certain optimal ND size at which the reflectivity reached a maximum. The latter depended on the geometric sizes of the reflector, its density and the number of impurities in the powder. However, increasing the ND diameter in the investigated range of parameters increases the reflectivity. Figure 11b shows that, as the ND size became larger, the angular distribution of the reflected neutrons became narrower. 

## 6. Analysis of Experimental Results

The common features of the observed QSR are the following:

1. The results in Figure 7 and Figure 9 and the performed simulations show that the angular spreads of QSR neutrons are significantly smaller for (F-)SCD samples than those for (F-)DND, which is explained by the smaller scattering angles on individual NDs.
2. The results in Figure 7 and Figure 8 show that, above the cutoff limit (~4.12 Å) for diffraction scattering on the crystal lattice of the diamond, the probability of QSR weakly depends on the wavelength, and below this limit, it drops sharply. This is due to the following reasons. Above this limit, the probability of QSR does not depend on the interaction of neutrons with the crystal structure of the diamond. It is determined by SANS on ND particles as a whole and by the neutron losses resulting from capture, inelastic scattering, incoherent scattering to large angles, the penetration of a part of the incident beam through the sample due to its finite volume, etc. Below the cutoff, neutrons scatter on the crystal lattices of NDs and experience intense scattering with large angles, leading to their loss from their QSR channel. Moreover, the probability of SANS by ND particles starts to rapidly decrease with decreases in the neutron wavelength. Thus, such neutrons can easily penetrate through the sample.
3. The much higher proportion of H in the DND and SCD powders compared to the F-DND and F-SCD powders results in a significantly lower probability of QSR. This is explained by the loss of neutrons due to incoherent scattering on H. This effect is most noticeable for short neutron wavelengths, because such neutrons penetrate deeper into the powder upon reflection.

In this work, we did not develop an exact quantitative description of all the features of QSR for all parameters of the problem due to its complexity and also due to the fact that the approach we chose seems sufficient for reliable qualitative conclusions and estimations. However, we would like to enumerate the conditions under which the exact quantitative description of the problem may become possible in the future, as follows: the inclusion of neutron diffraction on the crystal lattice; taking into account the structure, size distribution and small size of the ND itself; conducting a QSR experiment to detect all neutrons scattered in the 4-π angle (this option is not available for reflectometers); taking into account interference between simultaneous scattering on the shape of an ND and on its crystal structure; etc. 

## 7. Conclusions

As expected from our previous measurements of the QSR of neutrons from DND powders and from our model understanding of this process, the angular spread of reflected neutrons and the probability of QSR depend significantly on the size of the ND in the powder. We have experimentally shown that the angular spread of QSR neutrons is smaller when using larger NDs (SCD, ~15.0 nm) than standard DNDs (~4.3 nm). A comparison of our simulation with measurements showed that we can calculate with a high accuracy the QSR for neutrons with wavelengths greater than the diamond diffraction cutoff of ~4.12 Å. With larger ND sizes, the angular spread of reflected neutrons is narrower, a tendency that is valid up to much larger sizes. The QSR showed a maximum which corresponds to an optimal ND size, taking into account contributions from the geometric size of the reflector, its density and the number of impurities in the powder. Optimum ND diameters depend on the neutron wavelength and can be estimated as ~10–20 nm. The effect of the fluorination of larger NDs on reflectivity is important; thus, further increasing the efficiency of their fluorination is of particular interest. 

## Data Availability

Neutron data were obtained from experiments http://doi.ill.fr/10.5291/ILL-DATA.3-07-386, http://doi.ill.fr/10.5291/ILL-DATA.3-07-403, http://doi.ill.fr/10.5291/ILL-DATA.TEST-2772, http://doi.ill.fr/10.5291/ILL-DATA.EASY-977 with D17, D11 and PF1B [52] instruments at ILL, Grenoble, France.
